# A Disentangled VAE-BiLSTM Model for Heart Rate Anomaly Detection

**DOI:** 10.3390/bioengineering10060683

**Published:** 2023-06-03

**Authors:** Alessio Staffini, Thomas Svensson, Ung-il Chung, Akiko Kishi Svensson

**Affiliations:** 1 Precision Health, Department of Bioengineering, Graduate School of Engineering, The University of Tokyo, 7-3-1 Hongo, Bunkyo-ku, Tokyo 113-8655, Japan; alessio.staffini@bocconialumni.it (A.S.); tei@bioeng.t.u-tokyo.ac.jp (U.-i.C.); akiko-kishi@umin.ac.jp (A.K.S.); 2 Advanced Technology Department, ALBERT Inc., Shinjuku Front Tower 15F, 2-21-1, Kita-Shinjuku, Shinjuku-ku, Tokyo 169-0074, Japan; 3 Department of Economics and Finance, Catholic University of Milan, Largo Gemelli 1, 20123 Milan, Italy; 4 Graduate School of Health Innovation, Kanagawa University of Human Services, Research Gate Building Tonomachi 2-A 2, 3F, 3-25-10 Tonomachi, Kawasaki-ku, Kawasaki-shi 210-0821, Japan; 5 Department of Clinical Sciences, Skåne University Hospital, Lund University, 205 02 Malmö, Sweden; 6 Clinical Biotechnology, Center for Disease Biology and Integrative Medicine, Graduate School of Medicine, The University of Tokyo, 7-3-1 Hongo, Bunkyo-ku, Tokyo 113-8655, Japan; 7 Department of Diabetes and Metabolic Diseases, The University of Tokyo, 7-3-1 Hongo, Bunkyo-ku, Tokyo 113-0033, Japan

**Keywords:** heart rate, wearable devices, anomaly detection, deep learning, variational autoencoder

## Abstract

Cardiovascular diseases (CVDs) remain a leading cause of death globally. According to the American Heart Association, approximately 19.1 million deaths were attributed to CVDs in 2020, in particular, ischemic heart disease and stroke. Several known risk factors for CVDs include smoking, alcohol consumption, lack of regular physical activity, and diabetes. The last decade has been characterized by widespread diffusion in the use of wristband-style wearable devices which can monitor and collect heart rate data, among other information. Wearable devices allow the analysis and interpretation of physiological and activity data obtained from the wearer and can therefore be used to monitor and prevent potential CVDs. However, these data are often provided in a manner that does not allow the general user to immediately comprehend possible health risks, and often require further analytics to draw meaningful conclusions. In this paper, we propose a disentangled variational autoencoder (*β*-VAE) with a bidirectional long short-term memory network (BiLSTM) backend to detect in an unsupervised manner anomalies in heart rate data collected during sleep time with a wearable device from eight heterogeneous participants. Testing was performed on the mean heart rate sampled both at 30 s and 1 min intervals. We compared the performance of our model with other well-known anomaly detection algorithms, and we found that our model outperformed them in almost all considered scenarios and for all considered participants. We also suggest that wearable devices may benefit from the integration of anomaly detection algorithms, in an effort to provide users more processed and straightforward information.

## 1. Introduction

Cardiovascular diseases (CVDs) are a group of disorders that affect the heart and the blood vessels and cause millions of deaths every year [1,2]. A recent report [3] highlights that CVDs are the leading cause of death regardless of sex and ethnicity, and that one person dies every 34 s in the United States due to CVDs. Heart attack can also be “silent”, meaning that while the individual does not realise it has happened, damage has nevertheless occurred [4]. In addition, the economic burden associated with CVDs continues to increase [5,6].

Many studies use heart rate (HR) as the primary vital sign when trying to infer CVDs [7,8,9]. It is therefore of paramount importance to develop tools that inform individuals of possible anomalies (to be understood as unusual observations that do not conform with the expected HR pattern) in their HR behaviours, with the aim of preventing as much as possible the onset of CVDs [10].

The onset of CVD is difficult to predict and a conclusive diagnosis is challenging, as both depend on the idiosyncratic characteristics of the individual patient. Detecting small anomalies is a challenging task even for experienced physicians [11]. In recent years, researchers have focused on developing multiple approaches for predicting risk factors, such as logistic regression models [12,13], Cox proportional-hazards regression models [14,15], and accelerated failure time models [16]. A systematic review of prediction models for CVD risk can be found in the work of Damen et al. [17].

Machine learning models, for example random survival forests [18] and K-nearest neighbours algorithms [19], are also now providing interesting results. 

Nevertheless, none of these models can be applied interchangeably without any recalibration, due to different risk factor profiles among different regions and ethnicities [20,21]. The complex dependencies between risk factors and CVDs highlight a growing need to take into account the idiosyncrasies of individuals, to better prevent the onset of new cases and to better tackle existing cases.

Recent years have witnessed rapid diffusion in the use of wristband-style wearable devices. Prominent examples include the Fitbit, Apple Watch, and Garmin. These and others can monitor the user’s health and collect HR data [22]. Despite not being as accurate as a standard electrocardiograph (ECG) [23], these devices still provide much useful personalized information that can be used to identify irregularities or abnormal behaviours in vital data, for example HR anomalies. If properly processed, data obtained from wearable devices can accelerate the shift towards patient-centric care, which is a growing worldwide movement [24]. 

Anomaly detection is an active research field, with many applications in computer vision, manufacturing, finance, and medicine. It is concerned with the identification of observations that greatly differ from the majority of the dataset and from what can be considered a “normal” behaviour [25]. Not surprisingly, anomaly detection has also been extensively applied to wearable-device data. To name only a few such studies, Ref. [26] develops anomaly detection algorithms using scoring matrices extracted from wearables; Ref. [27] uses HR and sleep data obtained from Huami wearable devices to identify physiological anomalies caused by COVID-19 infection; Ref. [28] tests the ability of a smartwatch application to detect irregular pulses and identify atrial fibrillation; and ref. [29] compares multiple forecasting models on minute-by-minute HR data. A detailed review of anomaly detection for wearables data can be found in the work by Sunny et al. [24].

In the present study, we focus on HR values collected during sleep time using the Fitbit Versa (FV), a smartwatch produced by Fitbit Inc. We decided to focus only on sleep time for two reasons. First, the issue of missing values is a notorious problem when using data collected from wearable devices, which requires consideration of the most appropriate way to impute such values according to the situation at hand [24]. By including only participants who wore a FV during the night, we obtained continuous observations and thereby avoided the need to impute values and minimized the associated information loss. Second, anomalies in resting HR are known to be correlated with CVDs [30,31,32]; accordingly, by focusing on sleep time only, we minimized the noise in HR values introduced by individual peculiarities not clearly identifiable by the wearable device, such as alcohol consumption, physical activities, or anxiety/emotional distress. An important extension of this work (which is left for future research) would be to include other important HR predictors (such as activity level, effect of possible medications, and lifestyle habits) and also identify anomalies during the daytime.

In short, our contributions are as follows:

(1) We propose a combination of multiple unsupervised machine learning algorithms and a sliding-window-based dynamic outlier detection approach to label the data, taking into consideration both contextual and global anomalies.
(2) We develop an anomaly detection algorithm based on disentangled variational autoencoder (*β*-VAE) and bidirectional long short-term memory network (BiLSTM), and validate its effectiveness on HR wearables data by comparing its performance with well-known and state-of-the-art anomaly detection algorithms. Adding a BiLSTM backend to the VAE model allows us to capture contextual relationships in VAE-processed HR sequences by analyzing both forward and backward directions of the information flow. Ultimately, this leads the algorithm to better model the considered time series and to learn more accurate patterns.
(3) We explore the latent space of our proposed algorithm and compare it with that of a standard VAE, giving consideration to how tuning the *β* parameter helps with anomaly detection and with encoding temporal sequences.

A timely diagnosis is important to effectively combat heart rhythm disturbances [1]. With the introduction of wearable devices, HR tracking has become simpler, and it can be continuously performed [22]. Automatically identifying the presence of anomalies in HR alerts the individual about the possible presence of some CVDs, so that he/she can perform targeted clinical checks. An arrhythmia is an abnormality in the heart rate or rhythm, in which the heart can beat too fast (tachycardia, with a beat greater than 100 beats per minute (BPM)), too slow (bradycardia, with a beat below 60 BPM), or with an irregular rhythm. When the heartbeat is irregular, it is necessary to contact a medical doctor and, for more detailed information, a cardiologist [5]. Arrhythmia experts can do a lot in the treatment of HR disturbances, which might be a sign of the onset of CVDs such as myocardial infarction, arterial hypertension, and heart failure [11]. The treatment of cardiac arrhythmias is possible with both surgical and pharmacological treatment.

## 2. Materials and Methods

### 2.1. Study Participants and Wearable Device

The original participants of this study were recruited from five companies in Tokyo, Japan, as part of a larger project with the goal of studying the impact of lifestyle choices on metabolic syndrome (MetS). Participants were recruited from among employees who, based on the results of their annual health check-up, had been categorized with being at risk of MetS or having MetS. A total of 272 individuals were enrolled in a three-month randomized controlled trial on lifestyle changes. For the present study, as a first filter we focused only on individuals in the intervention group.

All participants received a FV as a smartwatch, together with instructions on how to wear it. Furthermore, they were asked to complete a questionnaire about their lifestyle, socioeconomic conditions, and past/present medical history. More detailed information on the FV and how it detects HR values can be found on the official company website (https://www.fitbit.com; accessed on 18 January 2023) and in our previous study [29].

Ethical guidelines and current regulations in Japan were respected in conducting the research. All participants were provided with detailed information about the original study and its purpose, and gave written consent for participation.

We selected eight participants (hereafter Participant 1, Participant 2, etc.) with heterogeneous characteristics and different lifestyles as a convenience sample to evaluate the performance of our considered models across a heterogeneous sample. Table 1 summarizes the differentiation in participant characteristics, including their age, sex, medical history, smoking/drinking habits, and exercise habits.

As previously mentioned, to reduce noise and avoid the need to impute values, we focused on sleep time only, and considered eligible only those participants who wore the FV during the night. In principle, the possibility of missing data remains due to instrument malfunctions or power loss [24]. However, we detected no missing values for any considered participant throughout the examination period.

Mean HR data were sampled both in 30 s and 1 min frequency scenarios.

For all participants and for every night, we excluded the first and last 30 min of sleep to remove possible irregularities in HR associated with falling asleep and waking up phases. 

### 2.2. Data Labeling and Preprocessing

Even if all the models considered in this study operate in an unsupervised manner and can be used, after training, for online anomaly detection, we needed to label the data to allow comparison of their performance in the test set. Data labeling for big data is known as a tedious and time-consuming task [33], and many anomaly detection algorithms have been proposed to automatically deal with the problem; for example, Ref. [34] has proposed the isolation forest (IF) [35] to create a labeled dataset, which is then used for training a long short-term memory (LSTM) autoencoder for land condition anomaly detection; Ref. [36] has applied the one-class support vector machine (OCSVM) [37] to identify anomalies in network traffic; and [38] has used clustering to separate anomalous log files from normal ones, and then fed the labeled data into an XGBoost [39] model to identify decision rules to perform classification. 

To our knowledge, however, no clear medical criteria to define anomalies in wearable-device data (as opposed to ECGs) have appeared, and no study has proposed how to automatically label them. To develop an effective detection algorithm, we want to consider both global and contextual anomalous HR values. First, we separately applied IF, OCSVM (with radial basis function kernel), and kernel density estimation (KDE) with a Gaussian kernel as anomaly detectors. Each of these methods has different pros and cons [40,41,42], such as with regard to the number of false negative errors, excessive sensitivity to noise, and detection of global anomalies only. To deal with these problems, we took the intersection of the sets of anomalies identified by each algorithm in an ensemble learning fashion. By doing so, we identified a set of points that can be considered anomalies with high confidence but are mainly global outliers. We therefore applied the sliding windows concept, selecting a window length of 3%, and classified as an anomaly any point three standard deviations above or below the HR mean of the considered segment. We then merged the set of points identified by the sliding window approach with the set of anomalies collected by intersecting the unsupervised machine learning methods; although some of the points (mainly global outliers) were present in both sets, doing so allowed us to add contextual outliers with greater confidence. Algorithm 1 schematically presents the data labeling process we utilized.
**Algorithm 1:** Data Labeling**Input**: HR data of the participant i ($HR_{i}$), set of participants I **Output**: set of anomalies for the participants (A) **Models**: Isolation Forest (IF), One-Class Support Vector Machine (OCSVM), Kernel Density Estimation (KDE), Sliding-Window (SW) **for** i = 1,2,…, I **do** $a_{i}\_IF$ ← $IF\left(HR_{i}\right)$ $a_{i}\_OCSVM$ ← $OCSVM\left(HR_{i}\right)$ $a_{i}\_KDE$ ← $KDE\left(HR_{i}\right)$ $a_{i}\_SW$ ← $SW\left(HR_{i}\right)$ $A_{i}$ ← {$a_{i}\_IF\cap$ $a_{i}\_OCSVM$ ∩ $a_{i}\_KDE$} ∪ $a_{i}\_SW$ **end for** A ← [$A_{1}$; $A_{2}$;…; $A_{I}$] **return** A 

After labeling, we split the data into training, validation, and test sets, considering 5 nights as training, 1 night as validation, and 1 night as testing. Our proposed anomaly detection algorithm and the other competitor models we introduce in the next section operate by being trained on “clean” (i.e., without anomalies) data. Therefore, we removed any anomalies from the training data by replacing their values with the previous non-anomalous HR values.

Table 2 reports the number of anomalies in the test set using both HR value scenarios, namely collection every 30 s and every minute.

### 2.3. Anomaly Detection Models

The main idea behind the anomaly detection models we implemented in this paper is as follows: first, we forecast values for the dependent variable (HR values) for a certain number of time steps; then, we measured the error between the predicted and true values and labeled those values whose error was above a selected threshold as anomalies. The threshold was defined in terms of the number of standard deviations from the mean of the squared errors over the training set; for each scenario we tuned this hyperparameter by evaluating the performance of the models on the validation set.

#### 2.3.1. ARIMA

Autoregressive integrated moving average (ARIMA) models are popular in time series forecasting [43,44,45] and have also been applied to anomaly detection [46,47]. 

The equation of $ARIMA\left(p,d,q\right)$ can be written as:

(1) $$\Delta_{d}y_{t}=\beta_{0}+\sum_{i=1}^{p}\beta_{i}\Delta_{d}y_{t-i}+\varepsilon_{t}+\sum_{j=1}^{q}\theta_{j}\varepsilon_{t-j},$$

where $\Delta_{d}$ denotes the d-th difference of $y_{t}$ (the dependent variable at time t), expressed as a linear combination of its p lagged observations and q lagged observations of the residual error terms. $\beta_{0}$ denotes the intercept of the ARIMA model, $B={\left(\beta_{1},\beta_{2},\ldots ,\beta_{p}\right)}^{T}$ and $\Theta ={\left(\theta_{1},\theta_{2},\ldots ,\theta_{q}\right)}^{T}$ are vectors of coefficients, and $\varepsilon_{t}\sim WN\left(0,\sigma^{2}\right)$. 

In practice, however, after applying the augmented Dickey–Fuller (ADF) test [48], we found that all our time series were stationary, so no differencing was required, and our formulation was equivalent to an $ARMA\left(p,q\right)$ model. Values of p and q were selected by observing the autocorrelation (AF) and partial autocorrelation (PACF) plots, whose specifications very often coincided with those provided by the Akaike information criterion (AIC) [49]. 

#### 2.3.2. LSTM

Long short-term memory (LSTM) [50] is a kind of recurrent neural network (RNN) which deals well with the vanishing gradient problem faced by the classical formulation of the latter [51]. It is suitable for sequence-prediction problems as it can capture long-term dependencies, making it also applicable to anomaly detection tasks [52,53]. LSTM networks comprise gates that control which information from the input should enter the network, be stored, or be discarded. The LSTM equations at a given time step t are as follows:

(2) $$F_{t}=\sigma \left(W_{F}x_{t}+U_{F}h_{t-1}+b_{F}\right),$$

(3) $$I_{t}=\sigma \left(W_{I}x_{t}+U_{I}h_{t-1}+b_{I}\right),$$

(4) $${\tilde{C}}_{t}=\tanh\left(W_{C}x_{t}+U_{C}h_{t-1}+b_{C}\right),$$

(5) $$O_{t}=\sigma \left(W_{O}x_{t}+U_{O}h_{t-1}+b_{O}\right),$$

(6) $$C_{t}=F_{t}\circ C_{t-1}+I_{t}\circ {\tilde{C}}_{t},$$

(7) $$h_{t}=O_{t}\circ \tanh\left(C_{t}\right),$$

where $F_{t}$, $I_{t}$, and $O_{t}$ denote the forget gate, the input gate, and the output gate, respectively; ${\tilde{C}}_{t}$ and $C_{t}$ are, respectively, the cell input activation vector and the cell status; $x_{t}$ is the input vector to the LSTM cell; and $h_{t}$ denotes the output of the cell. $W_{\cdot }$, $U_{\cdot }$, and $b_{\cdot }$ are, respectively, recurrent weights, input weights, and biases to be learned during training of the network; σ is the sigmoid activation function; and ∘ denotes the Hadamard product.

We implemented an LSTM anomaly detector similar to the one proposed by [52]. In particular, we stacked three LSTM layers (with 32, 16, and 8 cells, respectively), using a hyperbolic tangent (tanh) activation function. To prevent overfitting, we implemented max-norm regularization (bounding the norms of the weights and biases to be less than 3) and dropout [54] in each hidden layer. Adam [55] was used as optimizer, and early stopping was applied to further improve generalization performance [56]. 

#### 2.3.3. CAE-LSTM

Autoencoders (AEs) are neural networks that try to learn an efficient lower-dimensional representation of the input data. They are made by encoder and decoder components. First, the encoder compresses the input vector $x\in R^{n}$ into a lower dimensional embedding $z\in R^{d}$, with d<n; the decoder then tries to reconstruct x from z, producing an output vector $\tilde{x}\in R^{n}$. They have been used for dimensionality reduction [57,58,59], classification [60,61,62], and anomaly detection [63,64].

We implemented a convolutional autoencoder (CAE) coupled with an LSTM backend, following an idea similar to that described in [65]: first, the autoencoder was trained to learn good embeddings of the input data; then, we then passed these learned embeddings to an LSTM architecture like the one described in the above section. If the encoder generates meaningful embeddings, using them instead of the raw data allows the LSTM to track events over longer time windows.

We selected a CAE instead of a feedforward AE because it better reconstructed the raw samples. The encoder network was composed by three one-dimensional convolutional layers (16, 8, and 1 filter, all with stride lengths of 2 and with kernel sizes of 5, 5, and 1, respectively), with rectified linear unit (ReLU) activation function and stride length of 2. The raw samples to compress were of length n=32, and the latent space dimensionality d=4. The decoder network was a mirrored version of the encoder, which works well in practice [66]. Dropout and early stopping were again used as regularization techniques. With regard to the cost function C, which measures how good the decoder reconstructions were, we simply used the mean squared error (MSE) loss, i.e.,

(8) $$C\left(x,\tilde{x}\right)=||x-\tilde{x}{||}^{2}.$$

**Figure 1:**
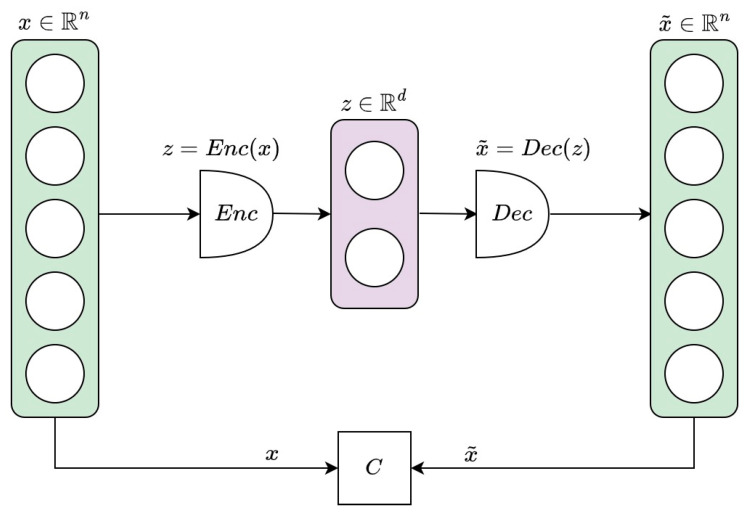
The structure of an autoencoder. An input vector $x\in R^{n}$ is first compressed by the encoder into a lower-dimensional representation $z\in R^{d}$. The decoder then tries to reconstruct x from z, producing an output vector $\tilde{x}\in R^{n}$. The autoencoder is trained to minimize the cost function C, measuring the discrepancy between x and $\tilde{x}$.

Figure 1 schematically illustrates the structure of the described autoencoder. 

#### 2.3.4. BiLSTM

Bidirectional long short-term memory (BiLSTM) [67] is an extension of the LSTM network and can be thought of as a combination of two LSTMs, one reading the input sequence forward and the second reading it backwards, after which their outputs are combined. By doing so we can increase the amount of information available to the network. BiLSTM proved itself particularly useful for sequential modeling tasks, such as text classification [68] and translation [69]. Recently, it has also been successfully applied in anomaly detection [70]. 

For a more meaningful comparison, we implemented a BiLSTM anomaly detector model with the same number of layers and cells per layer as its LSTM counterpart, described in the above section. Again, we applied dropout and max-norm regularization to prevent overfitting.

#### 2.3.5. *β*-VAE-BiLSTM

Variational autoencoders (VAEs) [71] are a probabilistic version of AEs, where instead of a compressed version of the input, the encoder outputs two parameters (the mean vector μ $\in R^{d}$ and the variance vector $\sigma^{2}\in R^{d}$), which describe the distribution of the input data over the latent space. Given μ and $\sigma^{2}$, we can sample a latent vector $z\in R^{d}$ using the reparameterization trick, which allows us to perform backpropagation during training:

(9) $$z=\mu +\sqrt{\sigma^{2}}\circ \varepsilon ,$$

with $\varepsilon \sim N\left(0,\;I\right)$. The VAE loss consists of two components:

(10) $$L=||x-\tilde{x}{||}^{2}+\beta D_{KL}(N\left(\mu ,\sigma^{2}\right)||N\left(0,I\right)),$$

where the first component is the reconstruction loss (as in AEs) and the second component, which acts as a regularization term, is the KL divergence between the encoded distribution and a standard Gaussian, with β denoting its strength. When β>1, we put a stronger weight on the KL loss, encouraging a more disentangled representation in the latent space [72]; this might help to create a more “regular” latent space, at the cost of a higher reconstruction error [73]. Due to their properties, VAEs have been applied both for data generation [74,75,76] and anomaly detection [77,78,79].

In this paper, we propose a β-VAE model coupled with a BiLSTM module, which we found helpful because reading the input sequence both forward and backward provided more context for the task. Inspired by [77], our approach is to first encode a vector of consecutive HR raw samples $x_{t}$ into the latent space using the β-VAE encoder; then, the BiLSTM module is trained over the obtained embeddings $z_{t}$ to predict the next embedding $z_{t+1}$; finally, the decoder decodes the predicted embedding ${\tilde{z}}_{t+1}$ into ${\tilde{x}}_{t+1}$, and by measuring its discrepancy with $x_{t+1}$ we label the anomalies. 

Other works have integrated unsupervised anomaly detection with forecasting; for example, SeqVL [80] uses a VAE for anomaly detection, followed by an LSTM module for trend prediction that operates on the decoded samples. However, unlike that case, we applied a BiLSTM module over the latent space embeddings and then decoded the predicted result, in line with [77]. Unlike [77,80], we found it effective to also use a BiLSTM structure (BiLSTM-VAE) in both the encoder and the decoder. More specifically, the encoder and the decoder were each composed of a single layer made of 64 BiLSTM units with tanh activation function. A possible drawback of using long windows is the delay in anomaly detection [77]; we tried to address this problem by shortening the window length and reducing the number of layers in the model, and we verified that (unlike the CAE-LSTM model) compressing samples of length n=10 into latent embeddings of dimensionality d=4 worked well empirically. Regarding the BiLSTM module trained to forecast the next embedding, we implemented an architecture similar (in terms of number of layers and number of units) to the LSTM described in the previous section. In most cases, we found it effective to provide as input only the current and the previous embeddings (i.e., $z_{t}$ and $z_{t-1}$) to forecast $z_{t+1}$.

In line with [79] and our empirical observations, we set β=3. 

**Figure 2:**
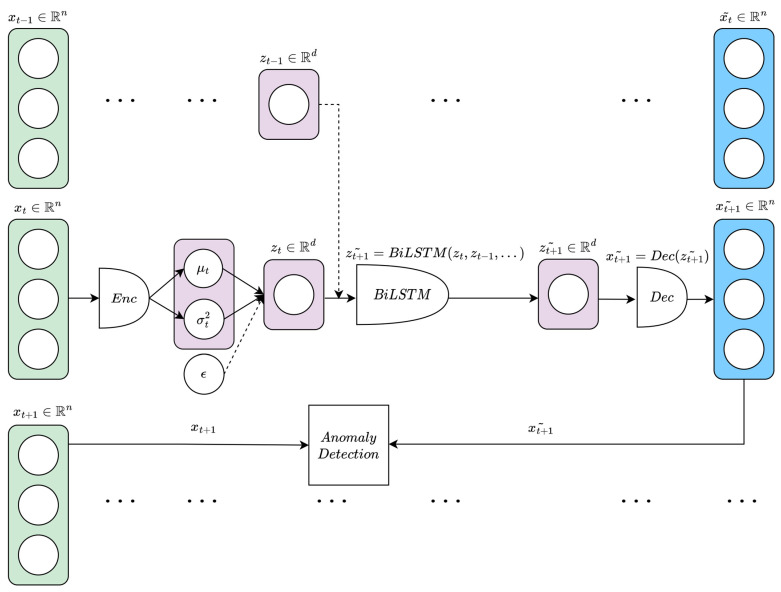
The structure of the proposed β-VAE-BiLSTM model. Non-overlapping input vectors $x\in R^{n}$ are compressed by the encoder into lower-dimensional representations $z\in R^{d}$, which are embedded in a more disentangled manner with respect to standard VAE, since we set β = 3. Then, the BiLSTM module takes as input the current embedding $z_{t}$ (and possibly previous embeddings $z_{t-1}$, etc.) to predict $z_{t+1}$. Finally, the decoder operates on the BiLSTM-forecasted embedding ${\tilde{z}}_{t+1}$, trying to reconstruct $x_{t+1}$ from it. We then measure the element-wise differences between ${\tilde{x}}_{t+1}$ and $x_{t+1}$, and label the anomalies accordingly.

Figure 2 shows the structure of our proposed model and how it operates.

## 3. Results and Discussion

We considered three metrics widely used in anomaly detection tasks to evaluate the performance of the models: precision, recall, and F1-score. Their equations are as follows:

(11) $$Precision=\frac{TP}{TP+FP}$$

(12) $$Recall=\frac{TP}{TP+FN}$$

(13) $$F1\;score=2\frac{Precision*Recall}{Precision+Recall}=\frac{2TP}{2TP+FP+FN}\;,$$

where TP, FP, and FN denote the number of true positives, false positives, and false negatives, respectively. Since in contiguous segments of anomalous values it is usually sufficient to receive an alert for the segment, instead of point-wise alerts, we augment the metrics with a strategy proposed in [81].

All the empirical analyses were conducted using Python 3.8.3 on an Anaconda platform (release: July 2020). The obtained results are reported in Table 3 and Table 4 for the 30 s and the 1 min frequency scenarios, respectively. Since neural networks have stochastic components in the training process (for example, the weight initialization), each algorithm was run 20 times for each scenario. Table 3 and Table 4 show the average mean of the runs, with the standard deviation in brackets.

From Table 3 and Table 4, we can observe that our proposed model performed significatively better than the competitor models in almost all the considered scenarios, regardless of participant or sampling frequency of the wearable device. ARIMA typically showed acceptable recall but low precision, meaning that many false alerts were often raised. LSTM and CAE-LSTM often performed closely and similarly, and we did not find any particular advantage in using the CAE encodings instead of the raw samples when forecasting the next time window. In general, both models tended to have acceptable precision and recall, and performed better than ARIMA. We found it useful to exploit bidirectionality to increase the context available to the anomaly detector model, as seen with the results of BiLSTM. Overall, our proposed β-VAE-BiLSTM model showed the best performance, typically displaying both good precision and recall, which in turn resulted in a balanced F1-score.

It should be mentioned that the anomalies of some users were more difficult both to define and to identify than those of other users (e.g., Participant 3), so all the models displayed relatively poor performance. In particular, we noted the difficulty in predicting anomalies in those who have more “unhealthy” lifestyles (i.e., frequent consumption of alcohol, smoking, and no physical exercise) and in those who have existing diseases. In line with existing studies, we noted that these participants are often characterized by increased sleep fragmentation (which negatively affects sleep duration [82]), reduction in sleep quality [83], and increased sleep disturbance [84]; as a consequence, their HR patterns exhibited greater variance and were usually noisy, which made it more difficult for the labeling algorithm to classify points as anomalies, and for the anomaly detection models to identify them. The obtained results further underline the already known importance of leading a healthy lifestyle.

With our proposed model, thanks to the wearable device, the individual is notified if he/she has had any anomalies in the previous time frame (i.e., 10 min or 5 min, depending on the sampling frequency). By doing so, an almost real-time response on the HR status is provided to the individual. Reports on the presence of anomalies suggest further clinical investigations, and are not intended as a diagnostic tool for CVDs. Our proposed model learns patterns from a “clean” (i.e., without anomalies) individual’s HR time series data, and signals as anomalies behaviours that are different from those learned.

Among the key strengths of this study, our focus on sleep only—a state which has fewer individual activities that are non-identifiable by the wearable device—allowed us to identify “true” HR anomalies with greater confidence. Furthermore, we considered the results provided by two different sampling frequencies for the same participants and compared and validated the proposed model with multiple competitor algorithms; furthermore, we analyzed both the distribution of the metrics produced by each model and how the different parameters influenced the calculated metrics. Finally, we validated the results obtained in multiple heterogeneous participants. 

This study has also some limitations that warrant mention. First, although abnormal values in resting HR are indicative of possible CVDs, we focused on a shortened period of time (night period only). Second, the models are applied to a single time series (resting HR), but it is important to consider the influence of other variables both in characterizing anomalies and in training the algorithms. Third, even if we tried to be comprehensive and consider heterogeneous participants, we considered only the factors listed in Table 1, and we could not control for some important lifestyle factors. Finally, the threshold used for anomaly detection was defined in a relatively simple way and could likely be improved.

By considering only measurements taken during sleep, we substantially reduced the variables that need to be considered. We are working to expand the data labeling algorithm and the proposed model to take into account other variables that can be detected by the wearable device, such as blood oxygen level, blood pressure, body temperature, and environmental temperature. Exploiting multivariate time series is likely to provide a more complete picture and consequently lead to better results, in both correct labeling and identification. A significant step forward would be to analyze also daytime data, by including in addition to the above-mentioned variables physical activity (a valid proxy could be the number of steps per minute) and weather data. 

### Temporal Embeddings in the VAE Latent Space

In this subsection we compare how the temporal sequences in the test set are encoded in the latent space of our trained β-VAE (with β=3) with respect to different β values, including a standard VAE model (i.e., with β=1). We conducted sensitivity analyses by setting β to 0.1, 0.5, 1, and 10 and, while the results did not vary much, we observed on average a slight deterioration in the metrics with respect to β=3 (see Table S1 in the Supplementary Material).

**Figure 3:**
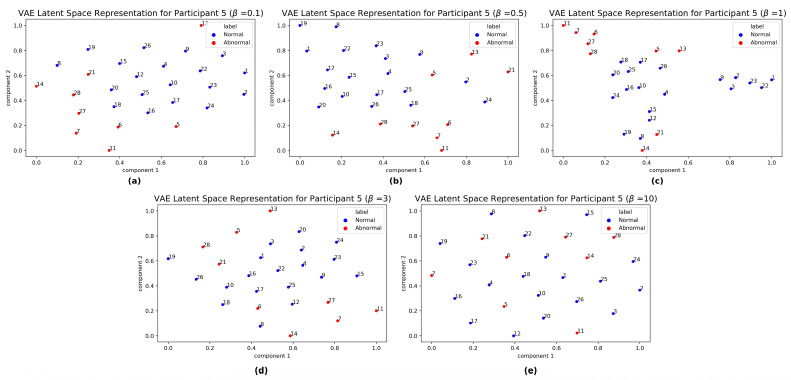
t-SNE maps for Participant 5. Projection of the latent space of the β-VAE model, with (**a**) β=0.1; (**b**) β=0.5; (**c**) β=1; (**d**) β=3; and (**e**) β=10. Numbering near the points denotes the order of temporal embeddings, each point being the embedding of a segment of length 10 of the 1 min frequency HR time series. Blue points denote normal test data (absence of anomalies in the original sequence), while red points denote abnormal test data (presence of anomalies in the original sequence).

Figure 3 shows the results of mapping in a two-dimensional space the temporal embeddings in the latent space of β-VAE (with different β values) for Participant 5 (1 min frequency), obtained by applying t-distributed stochastic neighbor embedding (t-SNE) [85]. The embeddings were scaled into the ${\left[0,1\right]}^{2}$ interval in order to remove the impact of the original scale. Similar mappings were also obtained for other participants (see Figures S1–S7 in the Supplementary Material). We used t-SNE instead of principal component analysis (PCA) because t-SNE better preserves the local structure of the data and because PCA is restricted to learn linear mapping.

We can notice how, as β increases, the latent clusters of normal and abnormal data become less dense and more spread out, in line with the observations in [86]. In principle, this behaviour is undesirable if we were to classify datapoints as anomalous or not directly from the embeddings in the latent space (for example, using a clustering algorithm). However, we argue that when passed in input to a BiLSTM backend, it is better to treat all the temporal embeddings as coming from the same distribution. In this way, the BiLSTM module is less affected by the possible presence of anomalies in the embeddings given as input and tends to output more “conservative” predictions which, combined with how we designed the detection system, ultimately results in better performance. However, t-SNE results are a non-linear mapping of the data into a lower-dimensional space, and should therefore be interpreted with caution. 

Different detection rules would likely provide different results, and we leave this comparison for future research.

## 4. Conclusions

In this paper we propose a disentangled VAE-BiLSTM architecture for unsupervised anomaly detection. We applied this architecture to heart rate data of multiple heterogeneous participants. Data were collected during sleep time with a wearable device, and we considered both 30 s and 1 min frequency scenarios. Empirical results showed that our model outperformed the competitor models we investigated in most cases.

To our knowledge, there is no consensus on the characteristics of anomalies in wearable data. To this end, we also proposed a simple but effective algorithm for automatic data labeling which takes into account both global and contextual anomalies.

Finally, we observed and compared the effects of how increasing the value of the β parameter affects the encoding of HR sequences into the latent space of VAE models. 

## Figures and Tables

**Table 1:** Characteristics of the selected participants.

Participant	Age(Decade)	Sex	PastDiseases	Present Diseases	Smoking/Drinking Habits	Exercise Habits
Participant 1	40s	Male	No diseases	No diseases	Past smoker; consumes alcohol 2–4 times per month	Exercises 3 or more days per week
Participant 2	50s	Male	2 diseases	1 disease	Smoker; consumes alcohol 2–4 times per month	Exercises 1–2 days per week
Participant 3	30s	Male	3 diseases	3 diseases	Smoker; consumes alcohol 4 or more times per week	No exercise
Participant 4	30s	Female	No diseases	No diseases	Non–smoker; consumes alcohol 2–3 times per week	Exercises 1–2 days per week
Participant 5	40s	Female	1 disease	No diseases	Non–smoker; consumes alcohol 2–4 times per month	No exercise
Participant 6	50s	Female	1 disease	1 disease	Non–smoker; consumes alcohol 4 or more times per week	Exercises 3 or more days per week
Participant 7	50s	Male	3 diseases	1 disease	Non–smoker; consumes alcohol 2–3 times per week	Exercises 1–2 days per week
Participant 8	50s	Female	2 diseases	No diseases	Non–smoker; consumes alcohol 1 time or less per month	No exercise

**Table 2:** Number of anomalies in the test set.

Participant	Number of Anomalies (30 s)	Number of Anomalies (1 min)
Participant 1	33	17
Participant 2	10	5
Participant 3	6	5
Participant 4	20	4
Participant 5	21	18
Participant 6	12	4
Participant 7	12	5
Participant 8	8	3

**Table 3:** Precision, recall, and F1-score of the tested models in the 30 sec frequency scenario. Values indicate the mean (standard deviation) of 20 runs for each model. Text in bold denotes the best results (95% confidence level) per participant.

Participant	Metrics	ARIMA	LSTM	CAE-LSTM	BiLSTM	*β*-VAE-BiLSTM
Participant 1	Precision	0.372	0.689 (0.073)	0.671 (0.048)	0.908 (0.032)	**0.949 (0.065)**
	Recall	**0.970**	0.628 (0.013)	0.688 (0.027)	0.656 (0.045)	0.715 (0.015)
	F1-score	0.538	0.655 (0.034)	0.678 (0.023)	0.761 (0.031)	**0.815 (0.029)**
Participant 2	Precision	0.162	0.800 (0.163)	0.812 (0.108)	0.732 (0.080)	**0.950 (0.068)**
	Recall	0.600	0.585 (0.036)	0.575 (0.043)	0.595 (0.022)	**0.611 (0.016)**
	F1-score	0.255	0.669 (0.063)	0.668 (0.034)	0.653 (0.045)	**0.742 (0.022)**
Participant 3	Precision	0.111	0.441 (0.199)	0.408 (0.083)	**0.517 (0.318)**	**0.625 (0.252)**
	Recall	0.167	0.208 (0.072)	0.275 (0.079)	0.300 (0.145)	**0.400 (0.111)**
	F1-score	0.133	0.265 (0.052)	0.315 (0.059)	0.319 (0.105)	**0.460 (0.115)**
Participant 4	Precision	0.351	0.708 (0.239)	0.875 (0.153)	**0.933 (0.220)**	**0.966 (0.073)**
	Recall	**0.650**	0.175 (0.097)	0.200 (0.100)	0.160 (0.110)	0.590 (0.080)
	F1-score	0.456	0.258 (0.156)	0.312 (0.114)	0.238 (0.181)	**0.727 (0.065)**
Participant 5	Precision	0.348	0.676 (0.120)	0.860 (0.043)	0.778 (0.041)	**0.915 (0.024)**
	Recall	0.762	**0.829 (0.038)**	0.769 (0.077)	0.765 (0.077)	**0.817 (0.017)**
	F1-score	0.478	0.738 (0.078)	0.809 (0.042)	0.768 (0.044)	**0.863 (0.014)**
Participant 6	Precision	0.174	0.712 (0.065)	0.760 (0.196)	0.745 (0.160)	**0.888 (0.124)**
	Recall	**0.333**	0.250 (0.000)	0.250 (0.000)	0.250 (0.000)	0.250 (0.000)
	F1-score	0.229	0.369 (0.010)	0.372 (0.023)	0.371 (0.019)	**0.389 (0.012)**
Participant 7	Precision	0.188	0.438 (0.006)	0.491 (0.032)	0.403 (0.047)	**0.** **747 (0.** **212)**
	Recall	0.500	0.658 (0.069)	**0.754 (0.104)**	0.667 (0.053)	**0.** **767 (0.081)**
	F1-score	0.273	0.525 (0.023)	0.591 (0.043)	0.500 (0.036)	**0.736 (0.105)**
Participant 8	Precision	0.219	0.694 (0.350)	0.843 (0.313)	**1.000 (0.000)**	**1.000 (0.000)**
	Recall	**0.875**	0.550 (0.061)	0.462 (0.057)	0.462 (0.057)	0.581 (0.006)
	F1-score	0.350	0.561 (0.180)	0.562 (0.142)	0.630 (0.056)	**0.** **733 (0.** **049)**

**Table 4:** Precision, recall, and F1-score of the tested models in the 1 min frequency scenario. Values indicate the mean (standard deviation) of 20 runs for each model. Text in bold denotes the best results (95% confidence level) per participant.

Participant	Metrics	ARIMA	LSTM	CAE-LSTM	BiLSTM	*β*-VAE-BiLSTM
Participant 1	Precision	0.448	0.722 (0.065)	0.610 (0.043)	0.792 (0.166)	**0.919 (0.069)**
	Recall	0.765	0.753 (0.024)	0.741 (0.073)	0.671 (0.073)	**0.806 (0.035)**
	F1-score	0.565	0.735 (0.035)	0.666 (0.038)	0.714 (0.081)	**0.857 (0.038)**
Participant 2	Precision	0.231	0.660 (0.073)	0.705 (0.069)	0.738 (0.054)	**0.938 (0.108)**
	Recall	**0.600**	**0.600 (0.000)**	**0.600 (0.000)**	**0.600 (0.000)**	**0.600 (0.000)**
	F1-score	0.333	0.627 (0.033)	0.647 (0.031)	0.661 (0.026)	**0.729 (0.036)**
Participant 3	Precision	0.333	0.466 (0.371)	0.268 (0.133)	0.548 (0.303)	**0.731 (0.203)**
	Recall	0.200	0.230 (0.145)	0.400 (0.000)	**0.560 (0.332)**	**0.610 (0.325)**
	F1-score	0.250	0.224 (0.186)	0.300 (0.106)	0.466 (0.174)	**0.611 (0.219)**
Participant 4	Precision	0.143	0.600 (0.184)	**1.000 (0.000)**	**1.000 (0.000)**	**1.000 (0.000)**
	Recall	**0.750**	**0.675 (0.225)**	0.540 (0.049)	0.538 (0.089)	0.682 (0.075)
	F1-score	0.240	0.570 (0.249)	0.700 (0.041)	0.695 (0.068)	0.809 (0.053)
Participant 5	Precision	0.478	0.800 (0.200)	0.813 (0.184)	**0.922 (0.073)**	**0.933 (0.037)**
	Recall	0.611	**0.903 (0.133)**	**0.853 (0.174)**	**0.825 (0.194)**	**0.872 (0.090)**
	F1-score	0.537	0.829 (0.124)	0.805 (0.116)	**0.857 (0.116)**	**0.898 (0.045)**
Participant 6	Precision	0.667	0.340 (0.143)	0.850 (0.166)	0.940 (0.092)	**0.990 (0.044)**
	Recall	0.500	**0.660 (0.358)**	0.500 (0.000)	**0.700 (0.245)**	**0.775 (0.249)**
	F1-score	0.571	0.406 (0.216)	0.624 (0.047)	**0.776 (0.154)**	**0.847 (0.169)**
Participant 7	Precision	0.278	0.748 (0.042)	0.825 (0.238)	0.736 (0.037)	**0.980 (0.060)**
	Recall	**1.000**	0.890 (0.099)	**0.970 (0.071)**	0.930 (0.095)	0.960 (0.080)
	F1-score	0.435	0.811 (0.054)	0.870 (0.158)	0.818 (0.038)	**0.968 (0.059)**
Participant 8	Precision	0.200	0.404 (0.344)	**1.000 (0.000)**	**1.000 (0.000)**	**1.000 (0.000)**
	Recall	**1.000**	0.667 (0.000)	0.500 (0.167)	0.467 (0.164)	0.667 (0.000)
	F1-score	0.333	0.436 (0.211)	0.650 (0.150)	0.620 (0.147)	**0.800 (0.000)**

## Data Availability

The authors cannot publicly provide access to individual data due to participant privacy in accordance with ethical guidelines. Additionally, the written informed consent obtained from study participants does not include a provision for publicly sharing data. Qualifying researchers may apply to access a minimal dataset upon reasonable request by contacting Saori Miyake at the following email address: miyake@bioeng.t.u-tokyo.ac.jp. The code of our model will be made available at: https://github.com/staale92/disentangled-vae-bilstm (accessed on 1 June 2023).

## Supplementary Materials

The following supporting information can be downloaded at: https://www.mdpi.com/article/10.3390/bioengineering10060683/s1, Table S1: Precision, recall, and F1-score of the *β*-VAE models. Values indicate the mean (standard deviation) of 20 runs for each model. Text in bold denotes the best results (95% confidence level) per participant; Figure S1: t-SNE maps for Participant 1; Figure S2: t-SNE maps for Participant 2; Figure S3: t-SNE maps for Participant 3; Figure S4: t-SNE maps for Participant 4; Figure S5: t-SNE maps for Participant 6; Figure S6: t-SNE maps for Participant 7; Figure S7: t-SNE maps for Participant 8.
