# Supplementary material for: A Disentangled VAE-BiLSTM Model for Heart Rate Anomaly Detection
Source: Bioengineering (Basel). 2023 Jun 3;10(6):683. doi: 10.3390/bioengineering10060683 (PMC10294855; doi:10.3390/bioengineering10060683)
Supplement: Supplementary file 1 [file bioengineering-10-00683-s001.zip › Figures S1-S7.pdf]

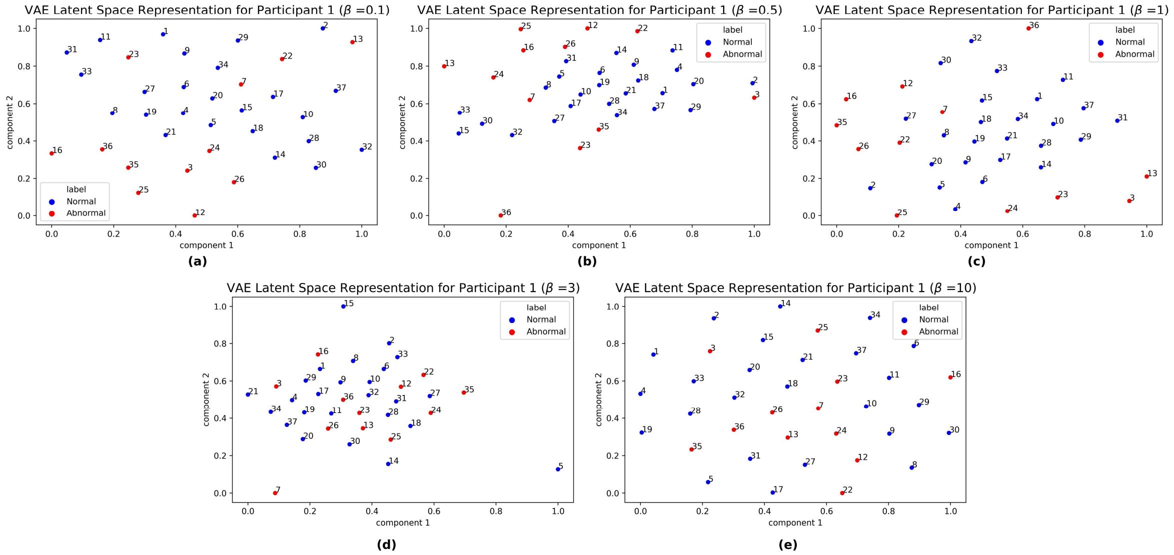

Figure S1: t-SNE maps for Participant 1. (**a**) $\beta = 0.1$; (**b**) $\beta = 0.5$; (**c**) $\beta = 1$; (**d**) $\beta = 3$; (**e**) $\beta = 10$.

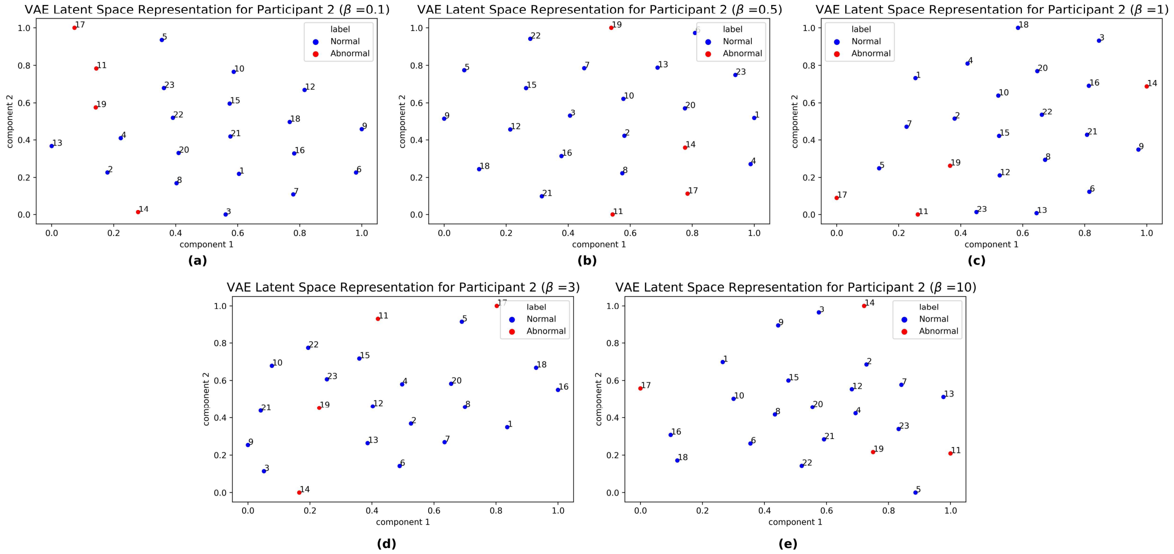

Figure S2: t-SNE maps for Participant 2. (**a**) $\beta = 0.1$; (**b**) $\beta = 0.5$; (**c**) $\beta = 1$; (**d**) $\beta = 3$; (**e**) $\beta = 10$.

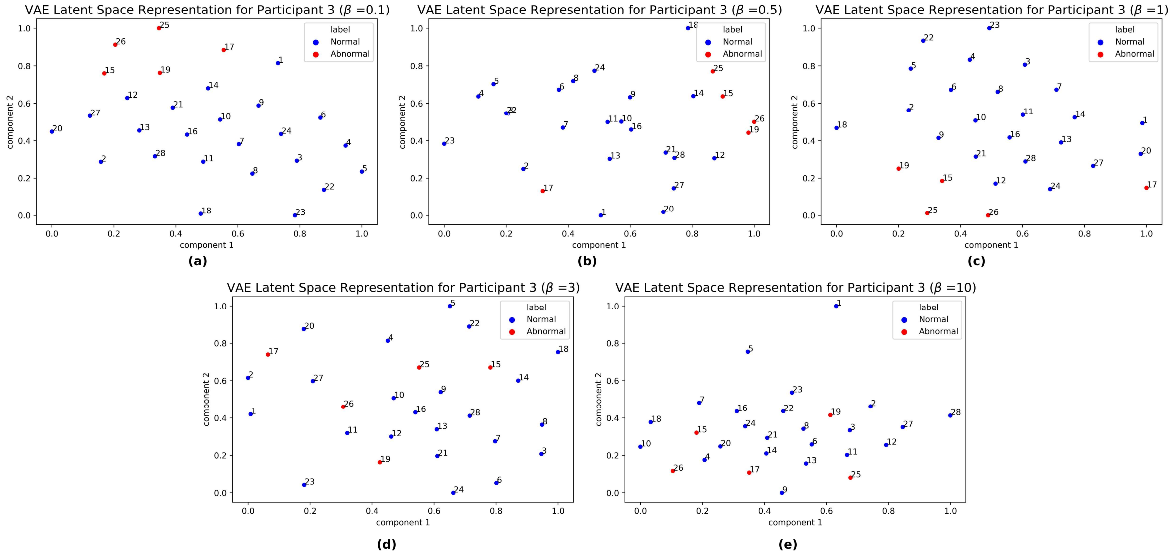

Figure S3: t-SNE maps for Participant 3. (**a**) $\beta = 0.1$; (**b**) $\beta = 0.5$; (**c**) $\beta = 1$; (**d**) $\beta = 3$; (**e**) $\beta = 10$.

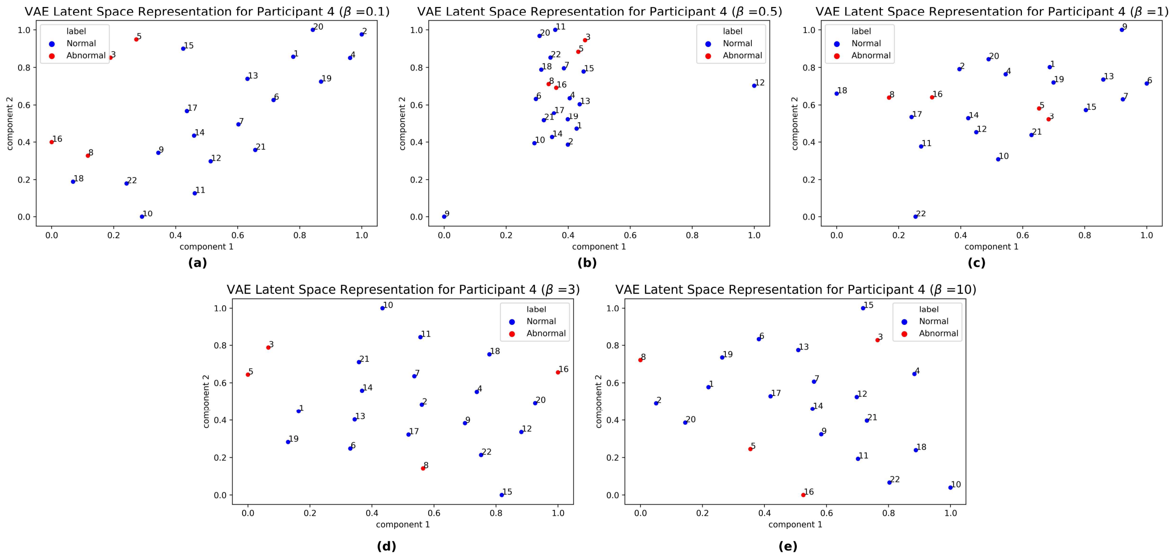

Figure S4: t-SNE maps for Participant 4. (**a**) $\beta = 0.1$; (**b**) $\beta = 0.5$; (**c**) $\beta = 1$; (**d**) $\beta = 3$; (**e**) $\beta = 10$.

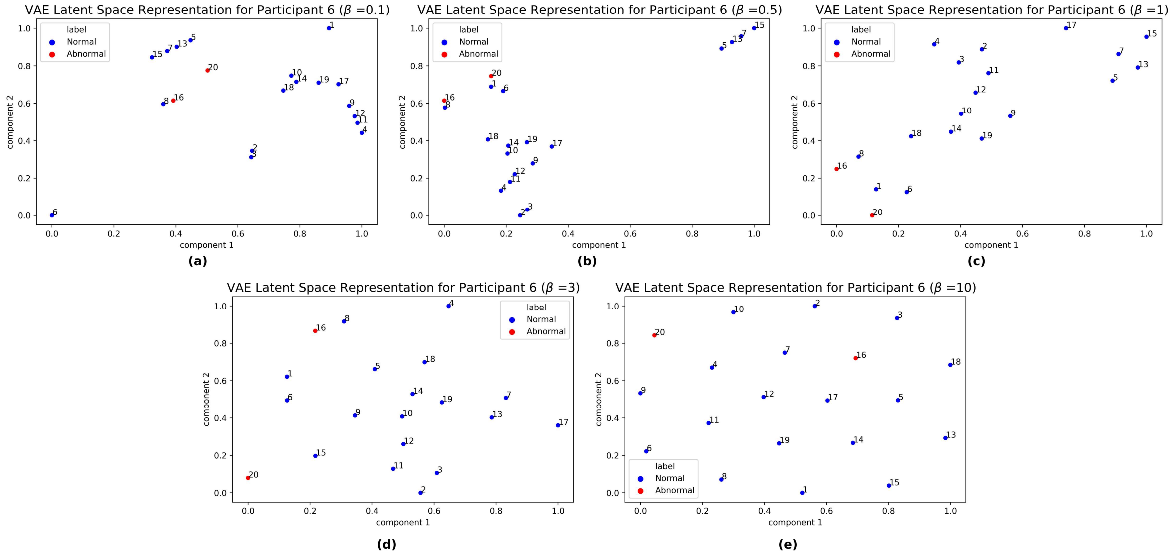

Figure S5: t-SNE maps for Participant 6. (**a**) $\beta = 0.1$; (**b**) $\beta = 0.5$; (**c**) $\beta = 1$; (**d**) $\beta = 3$; (**e**) $\beta = 10$.

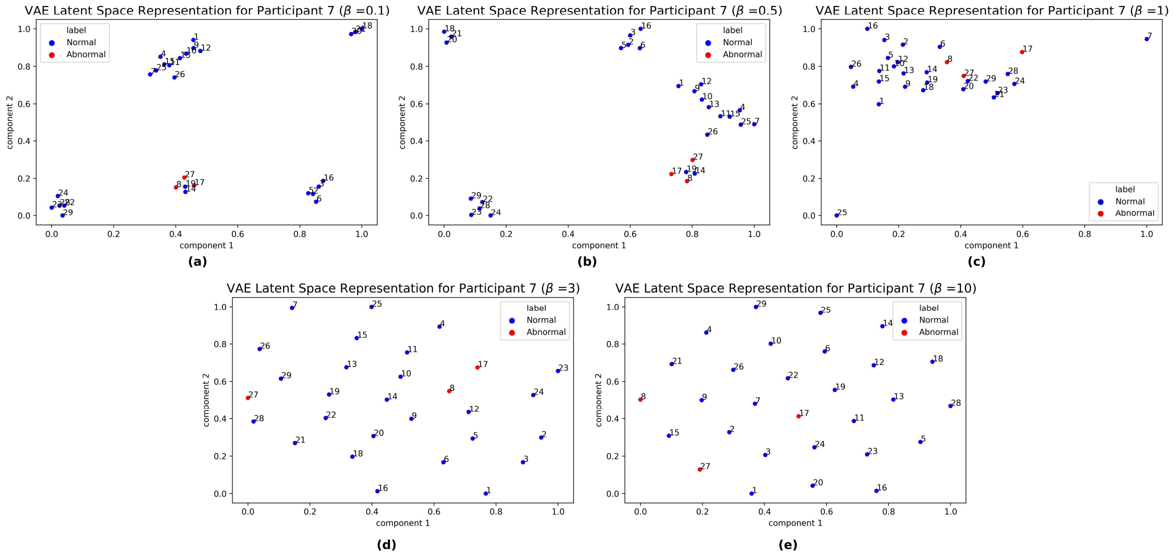

Figure S6: t-SNE maps for Participant 7. (**a**) $\beta = 0.1$; (**b**) $\beta = 0.5$; (**c**) $\beta = 1$; (**d**) $\beta = 3$; (**e**) $\beta = 10$.

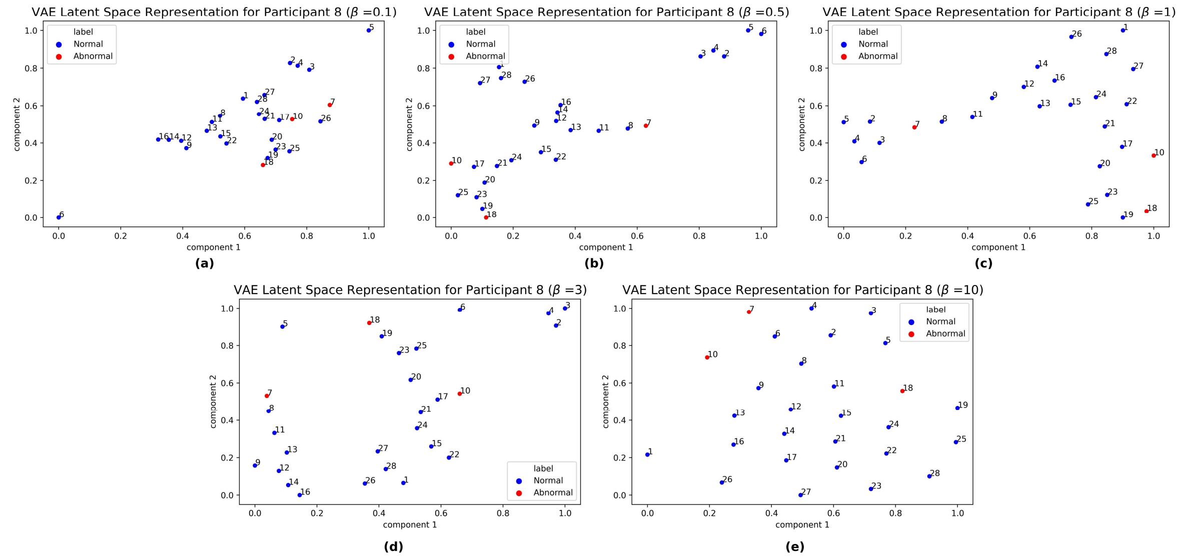

Figure S7: t-SNE maps for Participant 8. (**a**) $\beta = 0.1$; (**b**) $\beta = 0.5$; (**c**) $\beta = 1$; (**d**) $\beta = 3$; (**e**) $\beta = 10$.
