# Supplementary material for: A Disentangled VAE-BiLSTM Model for Heart Rate Anomaly Detection
Source: Bioengineering (Basel). 2023 Jun 3;10(6):683. doi: 10.3390/bioengineering10060683 (PMC10294855; doi:10.3390/bioengineering10060683)
Supplement: Supplementary file 1 [file bioengineering-10-00683-s001.zip › Table S1.pdf]

β-VAE-BiLSTM		β=0.1	β=0.5	β=1	β=3	β=10
Participant 1						
-	Precision	**0.879 (0.089)**	0.827 (0.081)	**0.866 (0.102)**	**0.919 (0.069)**	**0.895 (0.078)**
-	Recall	**0.780 (0.055)**	**0.783 (0.051)**	0.741 (0.054)	**0.806 (0.035)**	**0.815 (0.026)**
-	F1-score	0.825 (0.058)	0.801 (0.043)	0.796 (0.060)	**0.857 (0.038)**	**0.850 (0.034)**
Participant 2						
-	Precision	0.798 (0.175)	0.838 (0.119)	**0.880 (0.183)**	**0.938 (0.108)**	**0.900 (0.122)**
-	Recall	**0.600 (0.000)**	**0.600 (0.000)**	**0.600 (0.000)**	**0.600 (0.000)**	**0.600 (0.000)**
-	F1-score	0.677 (0.065)	0.696 (0.040)	**0.705 (0.069)**	**0.729 (0.036)**	**0.717 (0.041)**
Participant 3						
-	Precision	**0.775 (0.184)**	**0.744 (0.192)**	**0.662 (0.148)**	**0.731 (0.203)**	**0.762 (0.197)**
-	Recall	**0.670 (0.298)**	**0.590 (0.343)**	**0.720 (0.312)**	**0.610 (0.325)**	**0.700 (0.338)**
-	F1-score	**0.670 (0.178)**	**0.593 (0.232)**	**0.659 (0.204)**	**0.611 (0.219)**	**0.667 (0.237)**
Participant 4						
-	Precision	**1.000 (0.000)**	**1.000 (0.000)**	**1.000 (0.000)**	**1.000 (0.000)**	**1.000 (0.000)**
-	Recall	0.570 (0.046)	0.585 (0.036)	0.608 (0.093)	**0.682 (0.075)**	0.622 (0.102)
-	F1-score	0.725 (0.038)	0.737 (0.030)	0.752 (0.070)	**0.809 (0.053)**	0.762 (0.077)
Participant 5						
-	Precision	0.873 (0.115)	**0.903 (0.100)**	**0.906 (0.112)**	**0.933 (0.037)**	0.907 (0.016)
-	Recall	**0.822 (0.082)**	**0.856 (0.067)**	**0.831 (0.080)**	**0.872 (0.090)**	0.803 (0.010)
-	F1-score	0.842 (0.076)	**0.875 (0.066)**	0.860 (0.062)	**0.898 (0.045)**	0.848 (0.057)
Participant 6						
-	Precision	0.847 (0.135)	0.860 (0.147)	0.850 (0.144)	**0.990 (0.044)**	0.900 (0.129)
-	Recall	**0.850 (0.229)**	0.600 (0.200)	**0.725 (0.249)**	**0.775 (0.249)**	**0.750 (0.250)**
-	F1-score	**0.819 (0.134)**	0.692 (0.147)	**0.761 (0.172)**	**0.847 (0.169)**	**0.785 (0.148)**
Participant 7						
-	Precision	0.900 (0.100)	0.890 (0.099)	**0.930 (0.095)**	**0.980 (0.060)**	**0.905 (0.180)**
-	Recall	0.830 (0.071)	**0.930 (0.095)**	**0.910 (0.099)**	**0.960 (0.080)**	**0.910 (0.099)**
-	F1-score	0.860 (0.063)	0.904 (0.071)	**0.916 (0.076)**	**0.968 (0.059)**	**0.896 (0.126)**
Participant 8						
-	Precision	**1.000 (0.000)**	**1.000 (0.000)**	**1.000 (0.000)**	**1.000 (0.000)**	**1.000 (0.000)**
-	Recall	**0.634 (0.100)**	**0.667 (0.000)**	**0.667 (0.000)**	**0.667 (0.000)**	**0.667 (0.000)**
-	F1-score	**0.770 (0.090)**	**0.800 (0.000)**	**0.800 (0.000)**	**0.800 (0.000)**	**0.800 (0.000)**
